# The early infection characterization of septic arthritis by *Staphylococcus aureus* after anterior cruciate ligament reconstruction in a novel rat model

**DOI:** 10.1186/s13018-023-03969-1

**Published:** 2023-07-22

**Authors:** Kai Tong, Jian Wei, Zilin Liu, Xiaoming Yang, Yong Hu

**Affiliations:** 1 Department of Orthopedics, Renmin Hospital of Wuhan University, 238 Jiefang Road, Wuchang District, Wuhan, 430060 China; 2 Department of Joint Orthopedics, Liuzhou People’s Hospital, Liuzhou, China

**Keywords:** Anterior cruciate ligament reconstruction, Infection, Animal model, *Staphylococcus aureus*, Bacterial colonization

## Abstract

**Background:**

The present study aimed to explore the time of maximum bacterial load and main colonization knee site in bacterial infection process based on a novel rat model of septic arthritis (SA) after anterior cruciate ligament reconstruction (ACLR).

**Methods:**

Ninety-five Wistar rats with unilateral ACLR, random enrolled into control surgery (CS) group; joint inject (JI) group; presoaking (PS) group, were injected with 30 μl sterile saline or 30 μl × 10^7^ colony forming units/ml *Staphylococcus aureus* via the knee joint or graft with presoaked *Staphylococcus aureus* during ACLR, respectively. At 1, 4, 7, 11, and 14 days postoperatively, samples were harvested to evaluate progress of knee joint infection by postoperative body weight, body temperature, knee temperature, knee width, scales of tissue damage, serum inflammatory markers, microbiological counting, microcomputed tomography (Micro-CT), digital radiography, magnetic resonance imaging (MRI) examination, and scanning electron microscopy (SEM).

**Results:**

No systemic infection was observed in all rats. Comparing with serum inflammatory markers, tissue scores of inflammatory reactions, bacterial counts in the CS group, these data were significantly elevated in the JI group and PS group. The bone mass around the bone tunnel was lower and the soft tissue of knee showed more obvious swelling on MRI in the infection groups than that in the CS group at 7 and 14 days postoperatively. *Staphylococcus aureus* clusters on the surface of screw and graft were observed in the infection group. The whole colony forming units of *Staphylococcus aureus* maintained a continuous upward trend peaking 7 and 11 days followed by a balanced curve in the infection groups. Bone and soft tissue were found to have more bacterial counts than graft and screws.

**Conclusion:**

This animal model effectively mimics the acute knee infection after ACLR. We found that the bacterial colonization exhibited the peak of acute infection between 7 and 11 days postoperatively, with the major bacteria loads found in the bone, soft tissue.

**Supplementary Information:**

The online version contains supplementary material available at 10.1186/s13018-023-03969-1.

## Highlights

1. In the current study, we develop a novel rat model mimicking the clinical features of SA undergoing ACLR.
2. This novel animal model could effectively mimic and characterize acute S. aureus infection after ACLR, which supports further study on infection prevention and treatment undergoing ACLR. Clinically, it could also provide theoretical basis for early diagnosis and further treatment of infection after ACLR to orthopedic surgeons.
3. The bacterial colonization exhibit the peak of acute infection between 7 and 11 days postoperatively, with the major bacteria loads in the bone, soft tissue.

**Supplementary Information:**

The online version contains supplementary material available at 10.1186/s13018-023-03969-1.

## Introduction

Rupture of anterior cruciate ligaments is a considerably common injury, which requires arthroscopic reconstruction [1, 2]. Septic arthritis following anterior cruciate ligament reconstruction (ACLR) was considered a rare complication with a prevalence from 0.14% to 2.6% [3–5]. Controversy exists regarding what is the optimal diagnostic and management strategies of septic arthritis after ACLR. However, no systematic description of the characteristics of bacterial colonization in the acute septic arthritis after ACLR was previously reported, which is quite critical for the diagnosis and treatment of such septic arthritis.

Intra-articular infection has potentially devastating consequences for the knee, leading to joint stiffness, graft failure and chondral damage, even permanent knee functional disability [5], which also fails the expectations and life quality of the patients [6]. The early diagnosis and treatment are widely recognized as key factors in avoiding these adverse consequences and shortening hospital days [5]. However, an early symptom such as low-grade fever or pain without persistent effusion often may be mistaken as a normal reaction after surgery without specific symptoms. Therefore, the exact diagnosis of SA is often made late, at an average of 3 weeks or even 2 months after ACLR procedure [7]. This subsequently leads to a delayed treatment resulting in failure of the graft and loss of the joint function [8, 9].

The poor outcomes of delayed diagnosis and treatment reflect a pressing need to develop a representative septic arthritis animal model that permits comprehensive analysis on characteristics of periarticular bacterial loads. Given the complexity of the interplay between the host immune system, tissue heterogeneity and wound bed components, animal models have gradually replaced in vitro experiments as the gold standard tool for various study involved in wounds [10]. Nevertheless, to our best knowledge, there is currently only an initial rat model established in our early research [11] that simulates septic arthritis after ACLR procedure without clarifying the early infection characterization of knee septic arthritis.

In the current study, we intended to develop a novel rat model mimicking the clinical features of septic arthritis after ACLR to provide the experimental basis for the postoperative clinical diagnosis and antibiotic management plan. Moreover, we present the model along with effective assessments of serum inflammatory markers, bone and soft tissue damage, histologic staining, radiographic scans, and bacterial loads both on the implant surface and within periprosthetic tissues. With the increasing arthroscopic reconstruction of ACL, this study may have potential implications for future experimental investigations of septic arthritis after ACLR.

## Materials and methods

### *Staphylococcus aureus (S. aureus)* strain preparation

*S. aureus* NCTC-8325 was used in our animal experiment. It was derived from a clinical isolate of a bacteremia patient in previous studies [12, 13]. Bacterial cultures were grown in Luria–Bertani (LB) medium (Sigma-Aldrich, United States). Briefly, bacterial strains were incubated overnight in LB broth at 37℃ with shaking at 220 rpm. By measuring the absorbance value at 600 nm and using the plate count method, the bacterial concentrations were verified. Colony forming units (CFUs) were estimated after overnight culture of plates with an incubator [14, 15]. The bacterial cultures were then diluted in phosphate-buffered solution (PBS) at optical density value 1.0 (OD600 approximately 1 × 10^8^ CFU/mL) [16].

### Study design and procedures

**Fig. 1:**
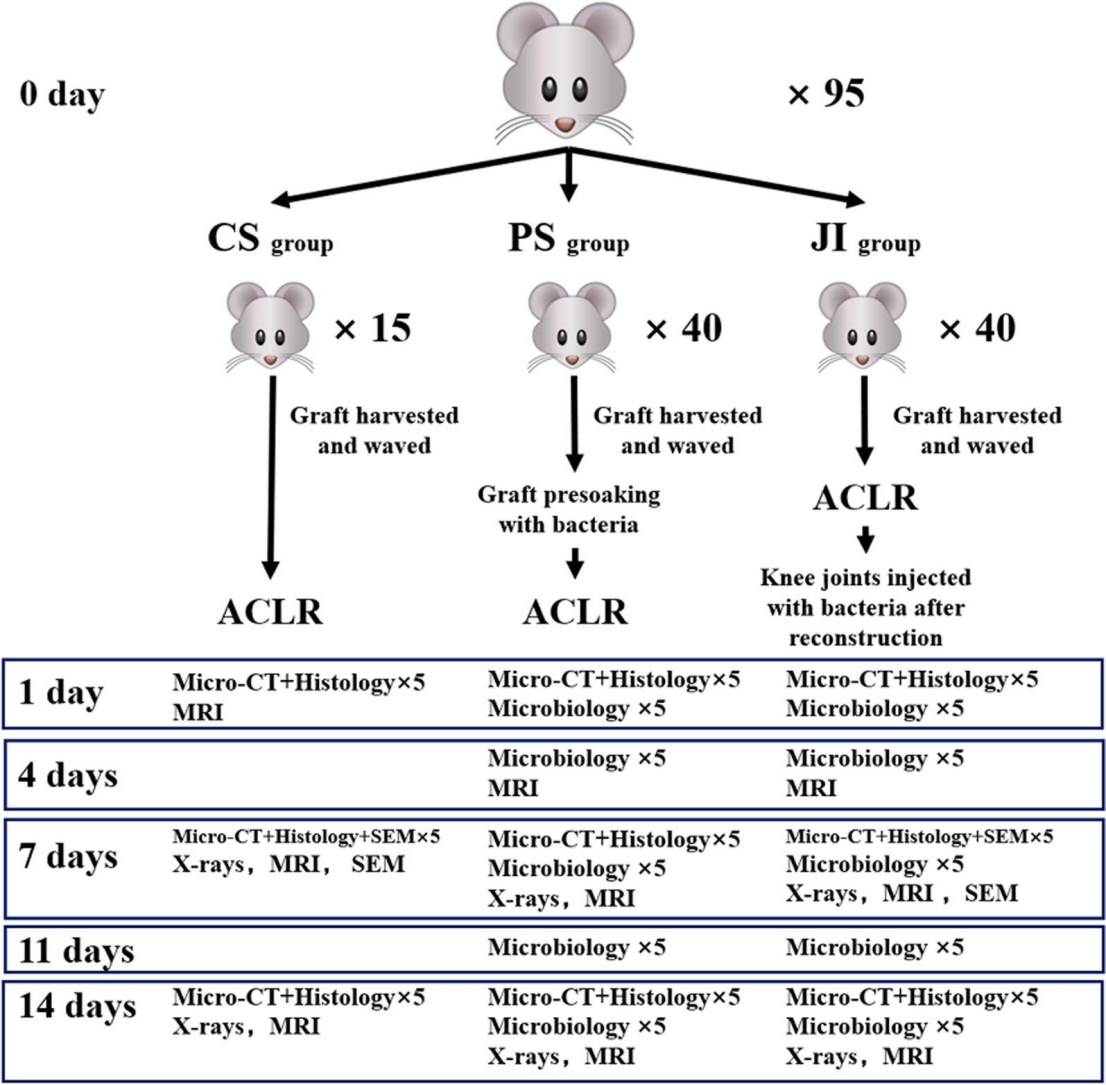
Summary of the experimental design. *ACLR* anterior cruciate ligament reconstruction; *CS group* Control surgery group; *PS group* Presoaking group; *JI group* joints injected group; *CT* computed tomography; *SEM* scanning electron microscopy; *MRI* magnetic resonance imaging

All animal experiments were approved by the Committee on the Ethics of Animal Experiments. Our animal experimental procedures were performed according to the Guide for the Animal Welfare Committee and Use of Laboratory Animals. The healthy Wistar rats of specific pathogen free (SPF) grade (male; weight, 280-300 g) were obtained and randomly allocated to 3 groups: Control surgery (CS) group, n = 15; Joint inject (JI) group, n = 40; Presoaking (PS) group, n = 40. These rats underwent unilateral ACL resection followed by ACLR according to an established method using an ipsilateral autograft peroneal longus tendon [17, 18]. Rats in the CS group only underwent primary ACLR, while these rats in the PS group utilized an ipsilateral autograft peroneal longus tendon with presoaking *S. aureus* before ACLR. Rats in the JI group underwent intra-articular injection *S. aureus* after ACLR. Further information of this study design is shown in Fig. 1.

### Surgical technique and bacterial inoculation

**Fig. 2:**
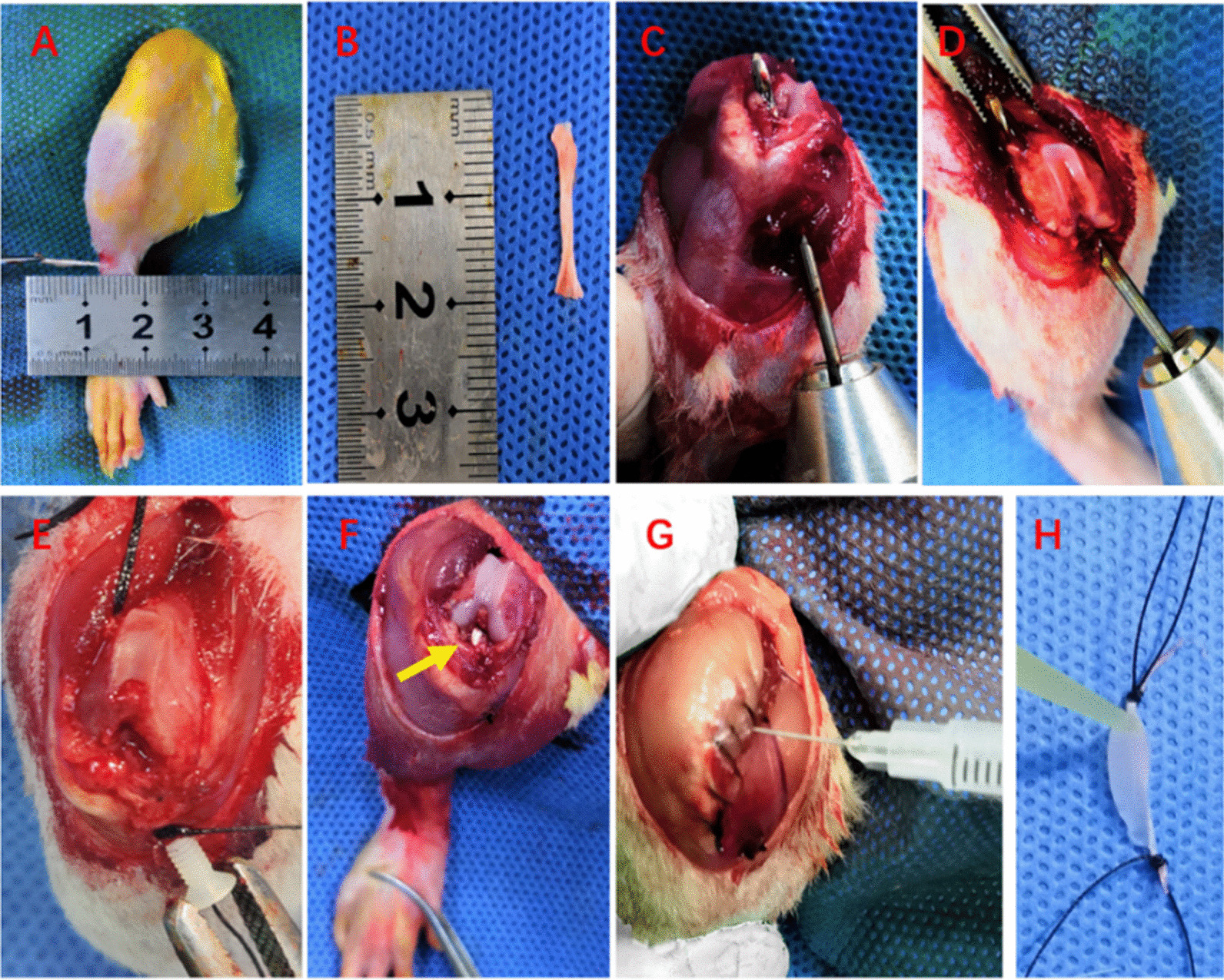
Surgical procedure for septic arthritis undergoing anterior cruciate ligament reconstruction (ACLR). **A**, **B** The right peroneus longus tendon was harvested and measured. **C**, **D** The bone tunnels of proximal tibia and distal femur were created using a 1.4 mm drill. **E**, **F** Both ends of the tendon graft were fixed by two 1.6 mm interference screws at the exit of the tunnel, and the yellow arrow showed the reconstructed graft. Infection was caused by intra-articular injection (**G**) or graft presoaking (H) of *S. aureus* solution

All rats were anesthetized with 2.5% isoflurane by inhalation delivered via nose cone and were given preoperative pain analgesics consisting of subcutaneous buprenorphine (0.1 mg/kg) and parecoxib (2 mg/kg). Routine cephalosporins antibiotic prophylaxis before surgery was given in all rats with a dose of 88 mg/kg in a rat, equal to 1 g in a patient weighting 70 kg [19]. After shaving, sterilization and draping, a percutaneous incision on the lateral side of the right ankle joint was performed and a 1.5 cm-2 cm length of the peroneus longus tendon of the right lower limb (Fig. 2A, B) was harvested with cleaning adherent muscle on the graft. And then, the lateral wound of the ankle joint was closed with 4–0/T nonabsorbable sutures (MERSILK SA83G, ETHICON, USA). The peroneus longus tendon autografts were weaved as single bundle with 3–0/T nonabsorbable sutures (MERSILK SA84G, ETHICON, USA) at both ends and wrapped in saline-soaked sterile gauze immediately in the CS group and the JI group. According to the results of the pre-experiment and a quantity depicted previously to successfully cause infection [20] before ACLR, those rats in the PS group utilized reconstructed grafts with presoaking bacteria with 30 μl × 10^7^ CFUs/ml for 10 min intraoperatively (Fig. 2H). A standard medial parapatellar arthrotomy was made into the right knee joint. The native ACL was identified and excised. The forward tibial drawer was applied to confirm the complete resection of the native ACL and the location of the tibia tunnel. Specifically, the bone tunnels of proximal tibia and distal femur were created using a 1.4 mm (diameter) drill (M1.4, BOSCH company, China) (Fig. 2C, D). The weaved autograft was introduced into bone tunnels and fixed by 1.6 mm (diameter) interference screws (M1.6, Guwanji company, China) (Fig. 2E) with the knee flexed to 30° pretensioned at a load of 5 N [17, 21, 22] (Fig. 2F). The wounds were closed with 4–0/T nonabsorbable sutures (MERSILK SA83G, ETHICON, USA) in the CS group and the PS group. To produce SA in the JI group, after the articular capsules were sutured, all rats received an injection of 30 μl × 10^7^ CFUs/ml bacteria with a 1.0 ml syringe needle (U40 0.3 × 8 mm, BBRAUN, China) (Fig. 2G), while these rats in the CS groups received 30 μl sterile saline. Then the wounds were closed (Fig. 2). Postoperatively, all rats received subcutaneous buprenorphine (0.05 mg/kg) for 24 h and were not restricted in physical activity.

### Sample collection and preparation

Postoperatively, all rats general condition and recovery of each group, including body weight, body temperature/knee skin temperature, knee width and wounds, were recorded and assessed multiple times before surgery and at five timelines of 1, 4, 7,11, and 14 days. An electronic thermometer (MC-246, OMRON, China) and an infrared thermometer (YT-1, Yuwell, China) for animals were used to measure the temperature of the anus and rectum and the local skin temperature of the surgical knee, respectively. An electronic vernier calipers (DL91150, Deli company, China) was used to measure the knee width of the surgical knee. By using the Rissing scale [20, 23], soft tissue and bone damage degree were quantitatively evaluated by wound erythema, bone destruction, and purulent exudate. Meanwhile, blood was collected before surgery and 4, 7,11, and 14 days postoperatively from 8 rats per group. After each rat was euthanized, the operative knees contained both whole bone tunnels were disarticulated by aseptic technique.

### Enzyme-linked immunosorbent assay (ELISA)

To elucidate severity degree of infection in each group at different time point, the blood samples were harvested for ELISA analysis. After anesthetized with 2.5% isoflurane, the jugular vein of the rat was cut, 5 ml venous blood of each rat was obtained with a centrifuge tube. And then the centrifuged supernatant was collected for the detection of circulating levels of α1-acid glycoprotein (α1-AGP), an acute phase reactant that rapidly upregulates within 1 to 2 days in response to infection or inflammation, especially in a rat [24]. The concentrations of serum α1-AGP were examined utilizing ELISA kits (AB157729, Abcam, USA) following the instructions of the manufacturer.

### Microbiological culture

The rats in the PS group and the JI group were sacrificed on postoperative day 1, 4, 7, 11, and 14 successively for bacterial culture. Sterile instruments were used to harvest tissue of same operated site and implant per rat, including the periarticular bone and soft tissues, graft, and screw. Before homogenized with a sterile tissue grinder, the tissues were cut into pieces and individually placed into a 5 ml EP sterile tubes of saline solution. Then, after vortexing, supernatant was plated onto LB agar petri dishes and grown with a continuous 37 ℃ incubator for 24 h. The screw was placed in a 5 ml EP tube containing 2 mL sterile saline solution and then sonicated (100 Hz) for 15 min in a water bath (37℃), to elute the bacteria on the surface of the screw. The supernatant was plated inoculated and grown on the LB agar petri dishes [20, 25–28]. After being confirmed as *S. aureus* by Gram staining, catalase testing and rapid agglutination testing of rabbit plasma coagulase, the number of bacterial colonies within the periprosthetic tissues was quantified using the plate count method [19, 29].

### Digital radiography acquisition

Digital radiography (Xtreme, Bruker, Germany) imaging system scanning, craniocaudal and lateral digital radiography of the operative knee were obtained to assess the function of knee joint at 7 days and 14 days postoperatively. All radiographs were assessed by 2 blinded observers.

### Micro-CT analysis

Carefully removing the interference screw, scanning of specimens was conducted perpendicular to the long axis of the lower limbs. Microcomputed tomography (Sky Scan 1276, Bruker, Germany) imaging system scanning, the parameters were adjusted as following condition [20, 25–28, 30]: resolution ratio, 35 mm; source voltage, 65 kV; source current, 380 mA and filter aluminum of 9 um. After scanning and data reconstruction, the same cross-sectional area of every bone tunnel in the tibia and femur was measured. A cylinder-shaped region of interest with a 1.8 mm height about 200 sectional area and 2.0 mm diameter was observed for analysis of the bone volume/total volume ratio (BV/TV). The BV/TV ratio was used to evaluate bone tunnel changes and the effects of bacteria on new bone formation in different groups.

### Magnetic resonance imaging (MRI) scans

After anesthetized, all animals were scanned in the prone position with an animal bed restraint system including ear bars and a bite bar for fixation of the head. Volumetric T2-weighted MRI scans of operative knees on a 7.0 T horizontal bore MRI testing instrument (Bio Spec 70/20USR, Bruker, Germany) equipped with self-shielded gradients capable of 600 mT/m with 125 ms rise times were acquired. A 3-dimensional Fast Spin-Echo (3DFSE) sequence with 150 mm isotropic voxel resolution was used to acquire the operative knee images and the following scan parameters: echo time (TE) = 35 ms effective, relaxation time (TR) = 2000 ms, flip angle = 90^◦^, matrix = 256 × 192 × 128, field of view (FOV) = 3.84 × 2.88 × 1.92 cm [31, 32]. After completing the T2 scanning, regions of interest (ROI) were placed manually on the region of the bone and soft tissue around the knee by referencing to the first-echo image of the T2 relaxation time. The ROI was defined as described in our previous report [32]. The shapes of the ROI were drawn by two experienced operators in knee MR imaging.

### SEM testing

Specimen of ligament and screw implants were harvested with soaking in the 2.5% glutaraldehyde solution. A field emission variable-pressure SEM (VEGA 3LMU, TESCAN, Czech Republic) was subsequently used to directly visualize biofilms on the intra-articular end and tunnel of the implants as previously described [25, 27]. The visual spherical structures of no surface deformities, organized in pairs or clusters, and approximately 1 μm in diameter was considered as the features of *S. aureus* [33]. Biofilm was defined as bacterial accumulation covered with an extracellular fibrin lattice or mesh. Host leukocytes were identified as spherical structures larger than 4 μm that were in proximity to bacteria, not covered by any extracellular material. Furthermore, the characteristic of host erythrocytes was typically a biconcave disk [20, 25].

### Histological evaluation

All samples of bone and soft tissue were next fixed in 4% paraformaldehyde, decalcified in a solution of 10% ethylenediaminetetraacetic acid, and embedded in paraffin. The specimens were sectioned coronally along with the long axis of the bone tunnel at a thickness of 5 um by a microtome (SM2500, Leica, Germany). Sections were stained with hematoxylin and eosin and gram staining. Per sample was calculated by averaging the width at whole tunnel’s cross section [17, 21]. The qualitative analysis performed by two independent observers. All images were photographed under an Olympus AH-2 light microscope (Olympus, Tokyo, Japan).

### Statistical analyses

All continuous variables were conducted for normality using the Shapiro–Wilk test. Parametric pairwise comparisons of two groups were performed using the *Student’s t-*test when appropriate. These data at different time point of multiple groups were compared by a two-way analysis of variance (ANOVA) or unpaired 1-tailed Mann–Whitney *U* test. Data were presented as the mean ± standard deviation (mean ± SD). All data analyses were performed with SPSS software (Version 22.0, IBM, USA). A *P* value less than 0.05 was considered significant.

## Results

### General status and serum inflammatory markers

**Fig. 3:**
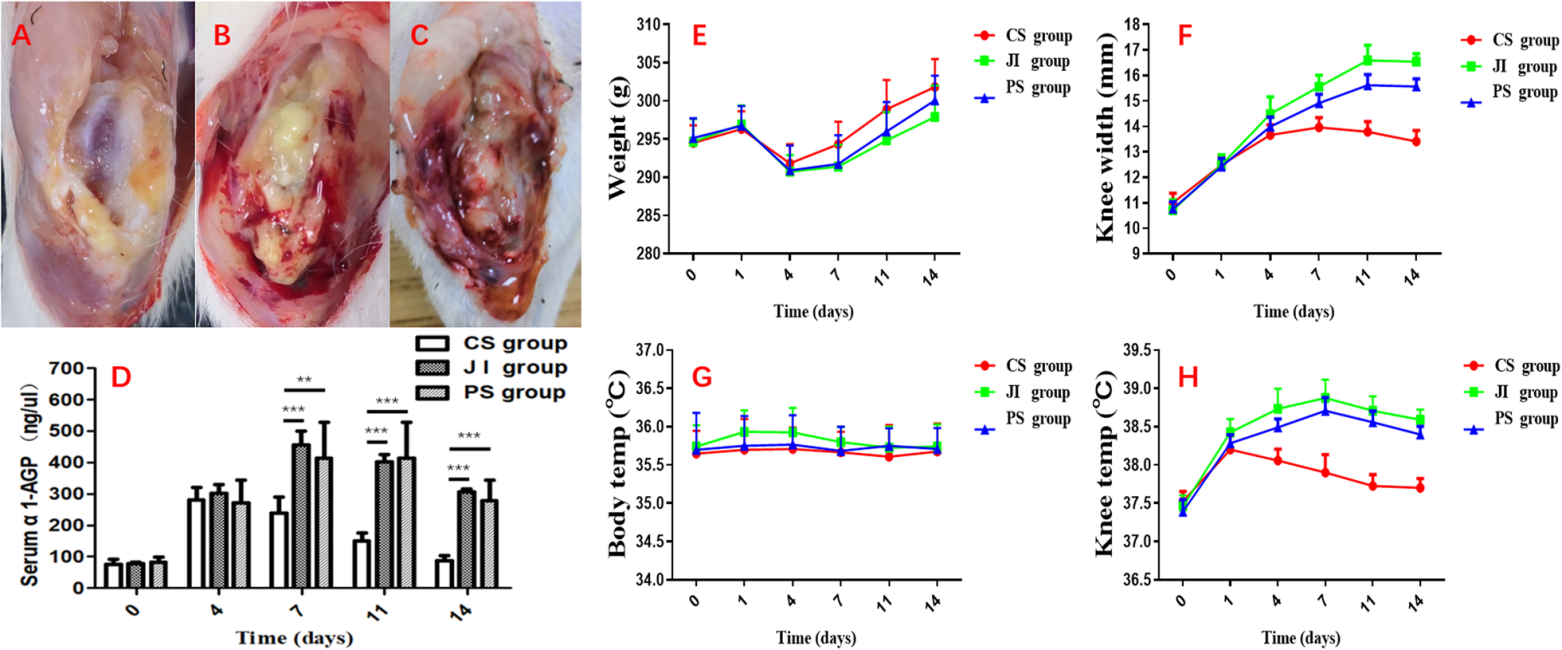
General condition after surgery. Representative image of right knee in the CS group (**A**), the JI group (**B**), and the PS group (**C**) at 7 days postoperatively. **D** Serum AGP level in different groups. Line charts showed mean rat weight (**E**), knee width (**F**), body temperature (**G**), knee temperature (**H**) in different groups over time. Knee width in the CS group was significantly smaller and lower than the JI group and PS group at 4 (*P* < 0.01, *P* < 0.05, respectively) and 7,11,14 days (*P* < 0.001, respectively). The data of knee temperature presented the same results. *CS group* Control surgery group; *PS group* Presoaking group; *JI group* joints injected group

No death occurred during the perioperative period. Obvious characteristics of infection approximate to clinical symptoms is shown in Fig. 3B, C. Bone destruction, distorted soft-tissue planes, and intra-articular purulent exudate were observed in the infection groups (JI group and PS group). The surgical wounds of knee remained closed and dry without draining or sinus. In addition, knee temperature has a significant difference appearing in the infection groups than CS group since 7 days postoperatively (Fig. 3H). Meanwhile, the knee width and body weight gradually varied wider and thinner in the infection groups than the CS group from 7 days postoperatively, respectively (Fig. 3E, F). Body temperature was not found significant difference between groups (Fig. 3G).

Those animals in the CS group, which exhibited preserved intact tibial plateau and femoral condyles, periarticular tissue planes, no purulent material (Fig. 3A) were significantly lower Rissing scale scores (1.50 ± 0.54) (*P* = 0.031) compared with that in the infection groups (JI group, 2.25 ± 0.71) at 4 days (Fig. 3B, C and Table 1). In fact, there was a bigger gap between CS group and infection group at 7, 11, and 14 days. The gross tissue score exhibited a decreasing trend in the CS group, but conversely an increasing trend in the infection group. Infected rats of both the JI group and the PS group had significantly elevated serum α-AGP levels at 7,11, 14 days postoperatively compared with controls (*P* < 0.01) (Fig. 3D).

**Table 1:** Gross tissue scale of all rats after ACLR

	Time of ACLR postoperatively (days)				
	1	4	7	11	14
CS group	1.25 ± 0.46	1.50 ± 0.54	0.63 ± 0.52	0.38 ± 0.52	0.13 ± 0.35
JI group	1.38 ± 0.52	2.25 ± 0.71 *	2.75 ± 0.89 ***	3.00 ± 0.93 ***	3.38 ± 0.74 ***
PS group	1.25 ± 0.46	2.00 ± 0.76	2.38 ± 0.52 ***	2.88 ± 0.84 ***	3.13 ± 0.64 ***

The gross tissue scale^31^ was determined by grading bone and soft tissue destruction from 0 to 4

0 score: absence of abscess, sequestrum, active bone formation, and erythema;

1 score: minimal erythema without abscess or evidence of new bone formation;

2 score: erythema with a widening of the head and shaft of the bone with new bone formation;

3 score: indicated abscess with new bone formation, sinus tract drainage, or grossly purulent exudate;

4 score: severe bone resorption, abscess, and diaphyseal or total tibial involvement

*P* value represents the comparison of the JI group and the PS group to the CS group, *P < 0.05, **P < 0.01, ***P < 0.001 (N = 5)

### Bacterial plate counts

**Fig. 4:**
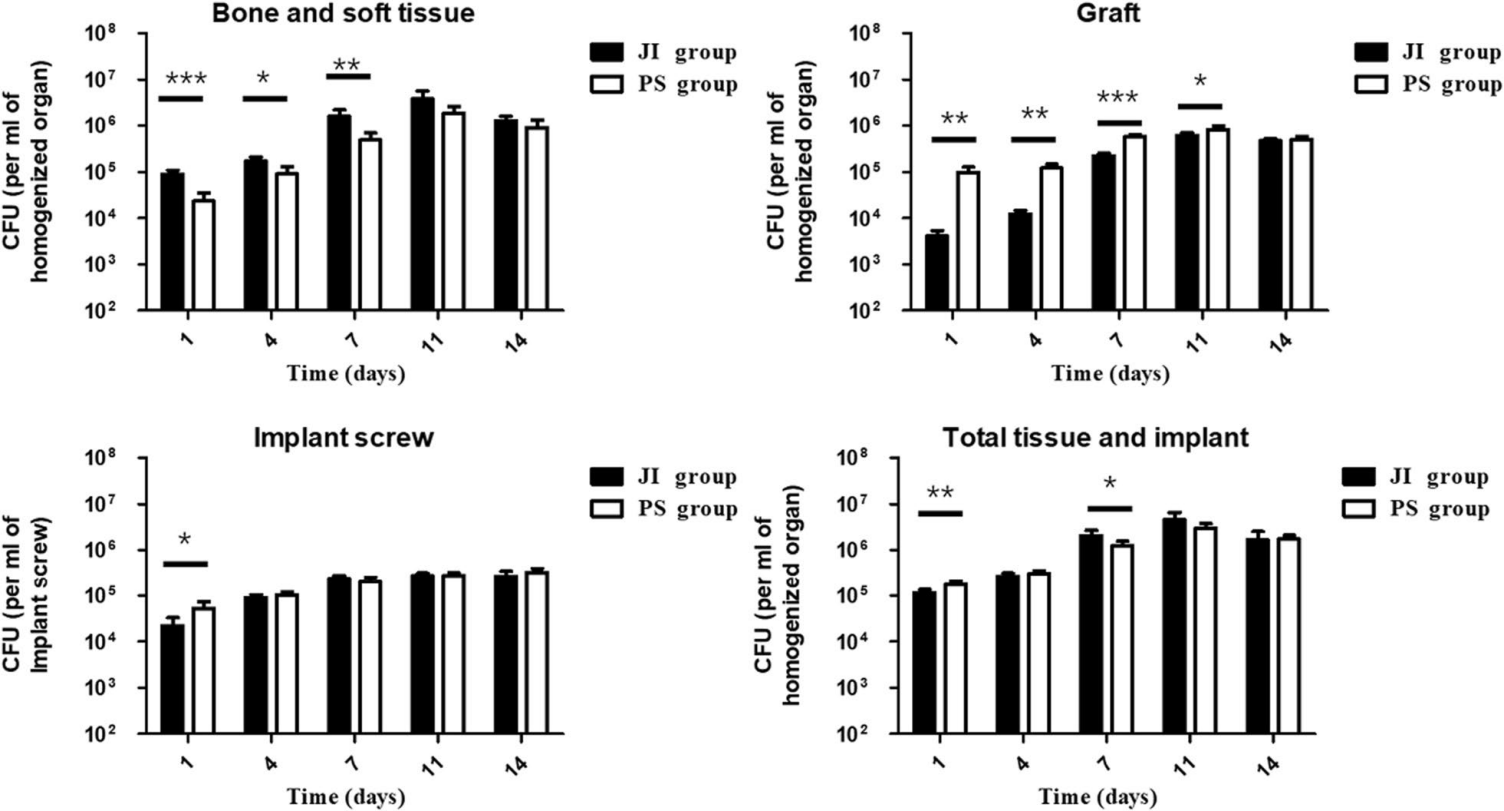
Bacterial counts of bone and soft tissues, graft, implant screw, and total tissue and implant from the infected rats among the JI group and PS group at 1, 4, 7, 11, and 14 days postoperatively. *P < 0.05, **P < 0.01, ***P < 0.001. *PS group* Presoaking group; *JI group* joints injected group

*S. aureus* were successfully retrieved, respectively, from bone/soft tissue, reconstructed ligament, and interference screws in the JI group and PS group. The bacterial plate counts covered all tissues in whole animal maintained a continuous upward trend from the postoperative 1 day to 7 days, which reach the peaking at 7 and 11 days followed by an equilibrium curve (Fig. 4). At 1 day postoperatively, bacterial counts increased approximately tenfold than those of the initial inoculations (1.15 ± 0.20 × 10^6^ CFUs, JI group and 1.76 ± 0.34 × 10^6^ CFUs, PS group). Bacterial counts in bone/soft tissues had gradually increased at 7, 11, and 14 days. The amounts of bacteria attached to bone and soft tissue, tendon, and interference screw were found decreased successively. There was no significant difference of bacterial counts on interference screws, implants, and total tissue between two groups at any time.

### Imaging (digital radiography, MRI, Micro-CT, SEM)

#### Digital radiography evaluation

Postoperatively, every interference screw was appropriately seated within the tibial and femoral canal without entering the articular aperture. At day 7 postoperatively, no obvious evidence of bone destruction and periosteal reaction had been noticed among all three groups. While the animals in the infection group (JI group and PS group) had bone damage and periosteal reaction at day 14. The representative digital radiography of craniocaudal and lateral position were shown in the Additional file 1: Fig. 1.

#### MRI evaluation

At 4, 7, and 14 days postoperatively, coronal MRI scans of the right knee were obtained with good signal-to-noise ratio. Cortical bone, trabeculae, screws, and graft presented dark signal on volumetric T2-weighted sequence images. Notch-like bright signal areas in the epiphysis of the femur and tibia were also noted on MR images. At 4 days, the MR images in the JI group showed the obvious high signal of periarticular soft tissue than the other two groups. Visually, no apparent difference was seen between the CS group and the PS group. Moreover, the brighter signal areas and the more extensive swelling images of soft tissue were found in the infection groups of 7 and 14 days than the controls. More details are shown in Additional file 1: Fig. 1.

#### Micro-CT evaluation

**Fig. 5:**
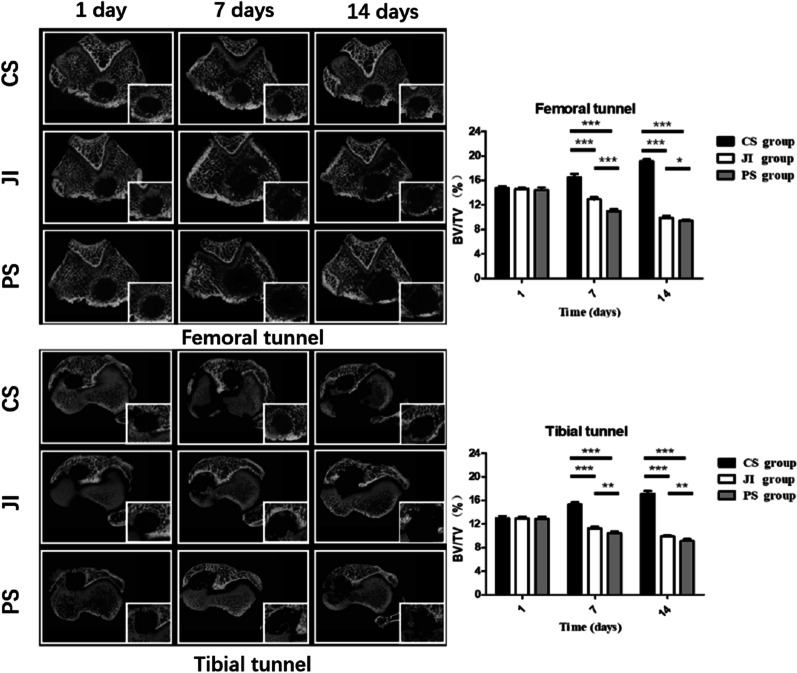
Relative tunnel area and the bone volume/total volume ratio (BV/TV) were evaluated by micro-CT at the femoral and tibial sides for ACLR at 1, 7, and 14 days postoperatively among the CS group, JI group, and PS group. Data are reported as ratios. *P < 0.05, **P < 0.01, ***P < 0.001. *CS group* Control surgery group; *PS group* Presoaking group; *JI group* joints injected group

Micro-CT evaluation showed the progress of bone tissue formation surrounding the graft within the tunnel and indirectly reflected the degree of infection. As regard to new bone formation, most bone tunnel regions in the CS group exhibited continuous increases in the BV/TV rate, but time-dependent decreases in the infection group from 1 to 14 days (Fig. 5). Among all 3 groups, bone tunnel regions in the CS group presented the smallest cross-sectional area, followed by the JI group and the PS group in sequence. For bone remodeling around the tunnel subregion, the average BV/TV rate was higher in the CS group than in the infection group on both the femoral and tibial side at 7 days investigated. As for the infection groups, there was lower value of BV/TV in the PS group than the JI group.

### SEM findings

**Fig. 6:**
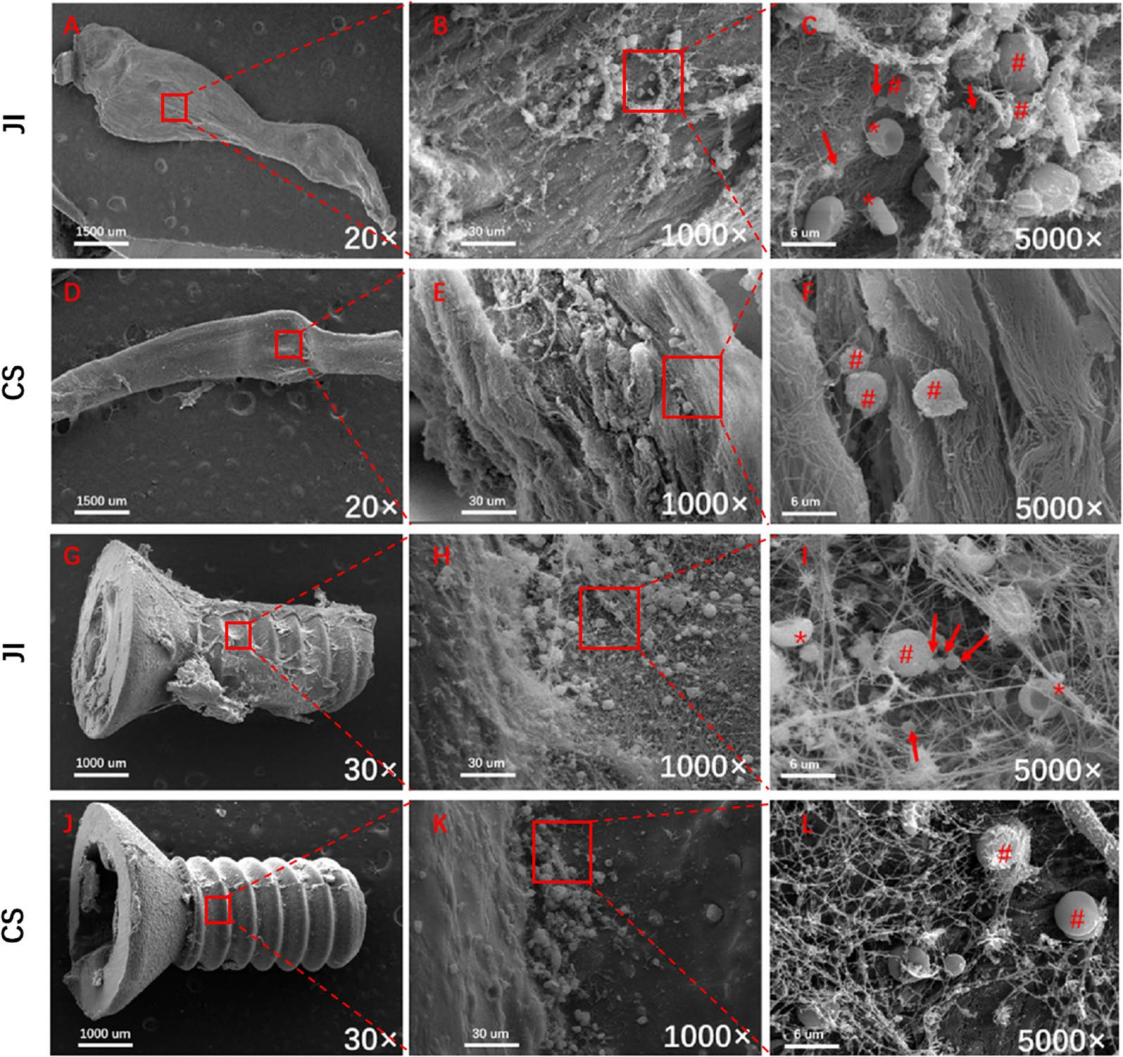
Scanning electron microscope (SEM) images of implants (Graft and Interference screw). Images of an infected graft and screw retrieved from an infected rat 7 days in the JI group (**A**, **G**) and CS group (**D**, **J**) were observed. Images of screw (**B**, **H**) with bacteria visible adjacent to the surface implant covered several fibrin-like shapes. **C** Clusters of *S. aureus* bacteria (arrows) surrounded by host leukocytes (pound signs). A red blood cell is also noted in the field (red asterisk) were noted. Magnified view in Fig. 7I showed clusters of *S. aureus* bacteria (arrows), host leukocytes (pound signs), red blood cell (red asterisk). A small amount of biofilm formation on the surface of screw and graft in the JI group. No bacteria were found covered the graft and screw in the CS group (**E**, **F**, **K**, **L**). *CS group* Control surgery group; *JI group* joints injected group. The part of red box was enlarged by the high magnification images

SEM examination of reconstructed ligament and interference screws from these infected rats in the JI group at 7 days postoperatively retrieved the presence of *S. aureus* clusters with abundant linear fibrin fibers connecting bacteria and densely packed within the walls of peri-implant (Fig. 6). A small amount of bacterial biofilm was found adjacent to screw (Fig. 6C) and graft surface (Fig. 6I). Some host leukocytes were observed on the screw and graft surface tissue with remaining *S. aureus* being engulfed by leukocytes. Meanwhile, host erythrocytes were also found on the screw and graft surface. In the CS group, there was no bacteria to be noticed covered the screw and graft. No bacteria were found covered the screw and graft in the CS group (Fig. 6E, F, K, L). Of all specimens on screw and graft, the amount of *S. aureus* was found more than 5 per 1 × 10^3^ or 5 × 10^3^ field of vision.

### Histological analysis

To evaluate degree of bacterial contamination of the periarticular knee and the graft in the bone tunnel, the inflammatory cell with hyperchromatic nuclei and a distinct graft-bone interface was observed in all 3 groups at 1, 7, 14 days. In the JI group and PS group, many inflammatory cells with hyperchromatic nuclei were identified than in the CS group. The relative width of the bone tunnel at 7 and 14 days was also significantly smaller in the CS group than in the JI and the PS group (1.50 ± 0.042 mm VS 1.66 ± 0.039 mm VS 1.70 ± 0.051 mm, *P* < 0.001 and 1.44 ± 0.036 mm VS 1.74 ± 0.044 mm VS 1.73 ± 0.055 mm, *P* < 0.001, respectively). Gram staining results showed that large number of bacteria attached to the cartilage in JI group and the graft in PS group at 7 days postoperatively but no bacteria in CS group. The histological results are reported in Additional file 2: Fig. 2.

## Discussion

In this study, we set PS group and JI group to simulate two different infection conditions clinically, namely the intraoperative graft contamination and surgical equipment contamination, by which, bacteria would be introduced into the knee to reproduce the hypothesized clinical etiology of septic arthritis [1, 34]. People have tracked the sedimentation of bacteria in 80 orthopaedic procedures, researchers found the rate of sedimentation varied from as low as between 48 CFU/m^2^/hour when large laminar air flow systems were used to 1870 CFU/m^2^/hour when smaller or less sufficient airflow systems were used. In cases where no laminar air flow was used, the rate was > 2000 CFU/m^2^/hour. Research shows that it takes approximately 1,000 bacteria contaminating a device to achieve close to a 100% device-related infection rate [35]. Acute infection following reconstruction of ACL is a rare but potentially devastating complication associated with bad functional outcome and increased morbidity [36, 37]. Clinically, staphylococci are one of the major pathogenic microorganisms found in up to 90% of septic arthritis after ACLR [36–39]. Multiple factors could cause complications of infection after ACLR, including contamination from the graft, implants for graft fixation, and surgical equipment [1, 34, 40]. However, there is no approximately clinical animal model to explain the characteristics of knee septic arthritis after ACLR. Overall results in this study should be used to clarify two questions, that is to present a novel animal model of *S. aureus*-induced septic arthritis after ACLR and to investigate characteristics of bacterial colonization and the process of bacterial infection development. Based on this model, more investigations on infection prevention and treatment undergoing ACLR can be accomplished. For example, how intraoperative vancomycin-soaking of the autograft with ACLR do prevent infection (based on the PS group rats) and what management strategies of postoperative infection after ACLR is at different time points (based on the JI group rats).

**Fig. 7:**
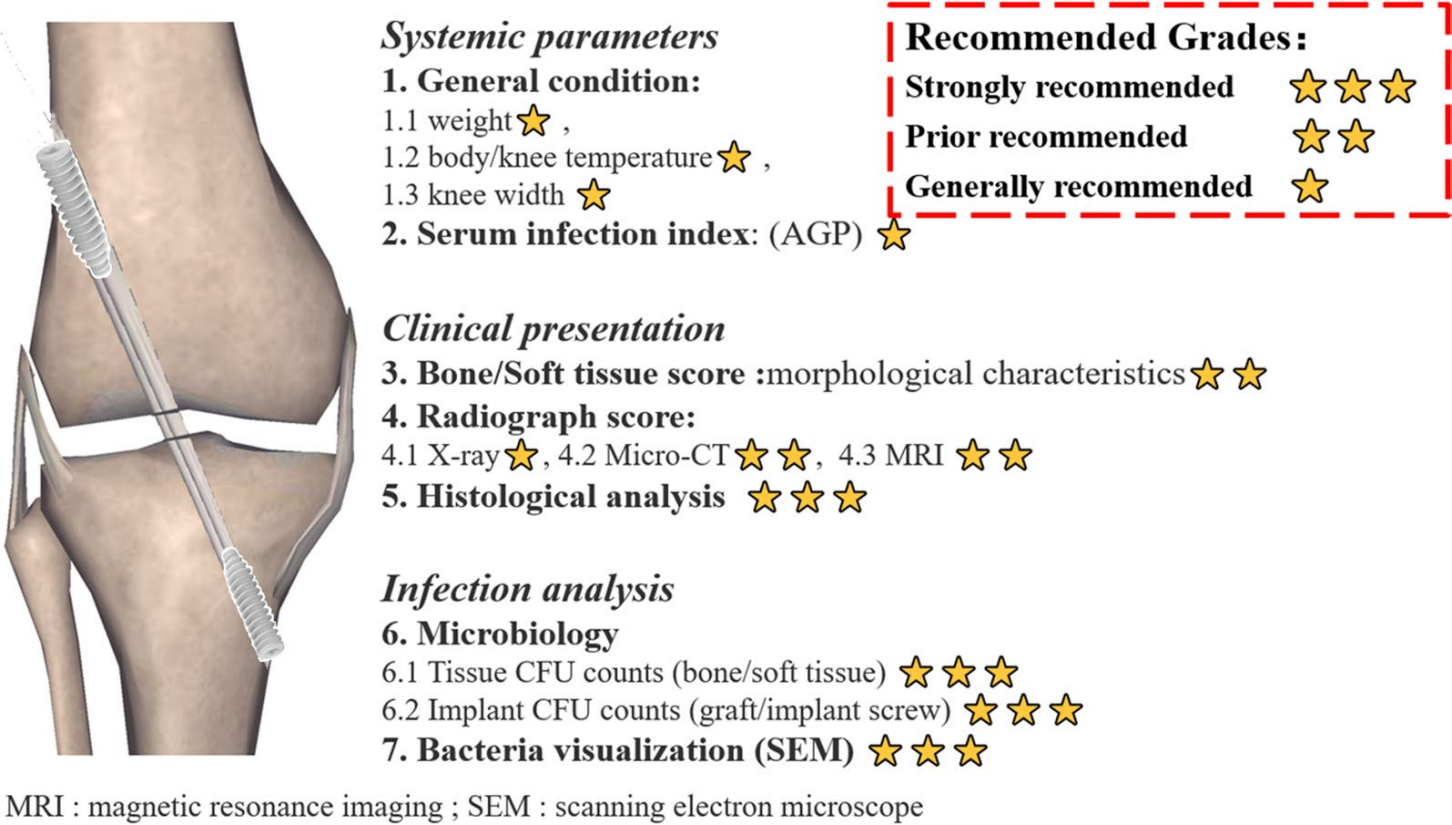
Summary of 7 parameters measured in the rat model of septic arthritis after ACLR

In the current study, widespread intra-articular purulence and distorted soft-tissue planes were noticed in the infection groups. Significantly higher Rissing scale score was obtained in animals of the JI or PS group were than in the CS group. Knee width, knee temperature, and serum α-AGP level in the infection groups exhibited sustained rise and maintained at a high level in accordance with the previous clinical studies [5, 7]. These indicator levels reached a level peaked at 4 days in the CS groups and gradually reduced. Thus, the symptom of persistent swelling and fever is not specific but should be paid special attention to. In addition, digital radiography, Micro-CT, MRI analysis, and SEM, microbiologic counts and histological study were depicted to definite the knee infection. Hereto, we presented a total of 7 aspects measures to evaluate septic arthritis undergoing ACLR (Fig. 7). Although this present study systematically presented a novel rat model of knee infection, there are important gaps between our animal model and clinical scenarios. Compared with the previous study [6, 41], except for the absence of the testing of synovial white blood cell count and special stains to evaluate articular cartilage, this current study provides more diagnostic criteria. Similarly, more animal model study of infection or wound could develop an advanced evaluation system based on our evaluation system in the future.

The outcome of digital radiography did not contribute sufficiently to early diagnosis of infection. It was only at 14 days postoperatively not early 7 days that bone destruction and periosteal reaction were found in the infected group. Of overall radiographic evaluation, new bone formation, tendon-bone healing interface, and bone remodeling around the bone tunnel could be observed using micro-CT, with indirectly reaction on serious degree of infection. Obviously, the BV/TV rate around the bone tunnel was lower in the infection groups than in the CS group. The sectional area of tunnel in the PS group was bigger than in the JI group at 7 and 14 days postoperatively. Expansion of bone tunnel and bone loss could lead to articular instability and poor tendon-bone healing, especially in the PS group. The published study by Wang et al. [17] also revealed that bone loss during infection progression is associated not only with *S. aureus* and its products, but also with inflammation. Further long-term studies are required to better elucidate tendon-bone healing suffering from septic arthritis.

We also noticed the phenomenon from microbiologic bacteria counts that the whole CFUs of *S. aureus* maintained a continuous upward trend to the peaking of 7 and 11 days followed by a balanced curve and the bacteria in the bone and soft tissue the presented the highest counts. In fact, the peak of local knee infection may be reached at 7 days postoperatively, when the inflammatory response of rat (such as body temperature, knee joint swelling, and serum infection indicators) is the most serious. While bacterial proliferation may gradually reach the upper limit at 11 days postoperatively, and then the CFUs of bacteria maintain a relatively stable. At this time, the body's systemic immune regulation and the infection will not continue to worsen. There may be a lag between the peak of bacterial proliferation and clinical symptoms. In this study, we found that the number of CFUs of S. aureus is higher in vascular bone and soft tissue than in avascular implants and graft tissue. There are two possible explanations for this phenomenon. At first, when a large number of bacteria colonized in the knee, the blood, soft tissue and bone tissue became a good culture medium for bacterial multiplication. Secondly, once the bacterium was placed into the knee and not cleared, with larger coverage area of bacterial exposure, bacteria implantation in soft tissues and bone tissues may be more extensive in short term than screws and grafts in bone tunnels. Therefore, we speculated that it happened probably acute infection peaks from 7 to 11 days and bacterial counts gradually became a balanced status without dynamical bacteria growth at 14 days according to bacterial plate counts of tissue and implant. The time point, especially from 7 to 11 days postoperatively and the infection site of bone, soft tissue should be paid more attention.

Several limitations in this study must be discussed. The dose of bacteria exposure occurring clinically is much lower than that the dose utilized in the infection groups [42]. It is an animal model specific limitation. In our previous pilot study, we found that rats have a stronger immune system than humans. The low doses of bacteria could not effectively happen knee infection and produce obvious symptoms of infection, while excessive bacteria may lead to death of rats. Finally, the appropriate dose of bacteria was selected by comprehensive assessment. However, I would be cautious to state that it emulates the clinical scenario in the human patient. A second limitation is that our surgery of ACLR is performed by an open surgical approach instead of arthroscopy clinically. A larger incision may lead to a higher risk of contamination and infection. However, almost all study of ACLR in small animal model was given an open operative approach due to a small size of knee [20, 33, 42]. In addition, due to a two weeks experiment without covering a healing time, there was no biomechanical data to support whether graft incorporation occurred clinically or not.

In conclusion, to our best knowledge, this present model effectively mimics the acute infection undergoing ACLR and has the potential to improve our fundamental knowledge of bacterial infection periarticular knee. Our study could provide a robust and affordable rat model to be used to evaluate the efficacy of diagnosis and antimicrobial treatments. We also depicted the characteristics and development of infection in our model, namely the peaking of acute infection between 7 and 11 days with the major bacteria loads on the bone and soft tissue.

## Supplementary Information

**Additional file 1: Figure 1**. Scans of digital radiography and MRI. (Left) craniocaudal and lateral radiographs of the rat knee at 7 days in the JI group. The red lines showed the bone tunnel footprint of proximal tibia and distal femur. (Right) coronal volumetric T2-weighted MRI images of the operative knee at 4, 7, and 14 days postoperatively in the CS group, JI group, and PS group. CS group, Control surgery group; PS group, Presoaking group; JI group, joints injected group.

**Additional file 1: Figure 2**. Gram stain and hematoxylin and eosin staining of operative rats after ACLR. (A) Bacteria colonized different parts of the knee at 7 days postoperatively among the CS, JI, and PS groups. The left red arrow indicates the articular cartilage and the right red arrow indicates the graft in bone tunnel Scale bar = 100 um. (B) representative image of the graft-bone interface at 1,7, and 14 days postoperatively among the CS, JI, and PS groups. The tendon-bone interface is outlined by the thick blue line. (C) statistical comparisons of the mean width of the bone tunnel. Scale bar = 200 um, B, bone; T, tunnel; G, graft. **—** represent 200 µm. CS group, Control surgery group; PS group, Presoaking group; JI group, joints injected group.

## Data Availability

We claim that all relevant raw data and materials in this study are available. We also agreed that all datasets on which the paper's conclusions rely should be available to readers.
