# Supplementary figures and images for: The early infection characterization of septic arthritis by Staphylococcus aureus after anterior cruciate ligament reconstruction in a novel rat model
Source: J Orthop Surg Res. 2023 Jul 22;18:522. doi: 10.1186/s13018-023-03969-1 (PMC10362564; doi:10.1186/s13018-023-03969-1)

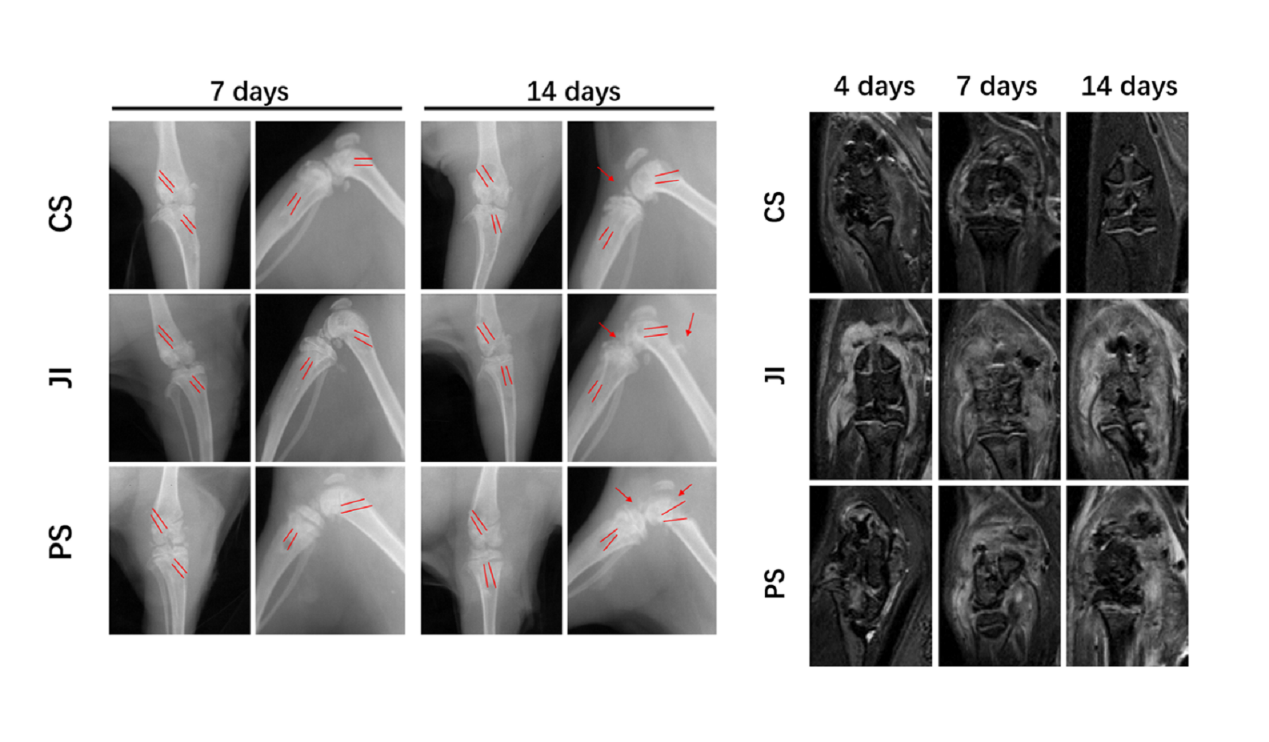


**Supplementary Figure 1**

Supplement: Supplementary file 1 — Additional file 1: Figure 1. Scans of digital radiography and MRI. (Left) craniocaudal and lateral radiographs of the rat knee at 7 days in the JI group. The red lines showed the bone tunnel footprint of proximal tibia and distal femur. (Right) coronal volumetric T2-weighted MRI images of the operative knee at 4, 7, and 14 days postoperatively in the CS group, JI group, and PS group. CS group, Control surgery group; PS group, Presoaking group; JI group, joints injected group. [file 13018_2023_3969_MOESM1_ESM.doc]

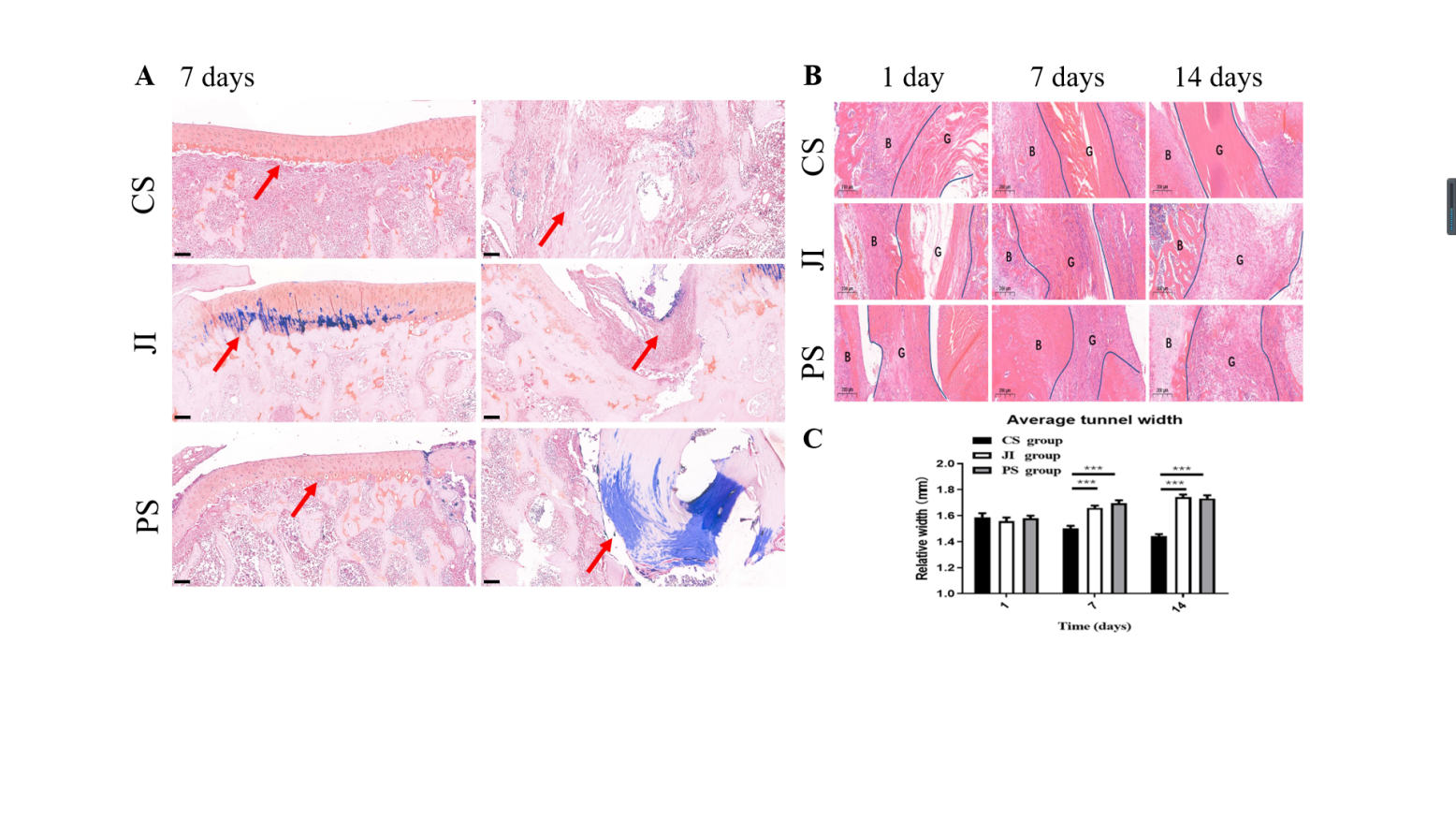


**Supplementary Figure 2**

Supplement: Supplementary file 2 — Additional file 1: Figure 2. Gram stain and hematoxylin and eosin staining of operative rats after ACLR. (A) Bacteria colonized different parts of the knee at 7 days postoperatively among the CS, JI, and PS groups. The left red arrow indicates the articular cartilage and the right red arrow indicates the graft in bone tunnel Scale bar = 100 um. (B) representative image of the graft-bone interface at 1,7, and 14 days postoperatively among the CS, JI, and PS groups. The tendon-bone interface is outlined by the thick blue line. (C) statistical comparisons of the mean width of the bone tunnel. Scale bar = 200 um, B, bone; T, tunnel; G, graft. — represent 200 µm. CS group, Control surgery group; PS group, Presoaking group; JI group, joints injected group. [file 13018_2023_3969_MOESM2_ESM.doc]
